# Harnessing the interaction between redox signaling and senescence to restrain tumor drug resistance

**DOI:** 10.3389/fcell.2025.1639772

**Published:** 2025-07-09

**Authors:** Hao Wu, Yang Yu, Xiangning He, Yanju Gong, Jianqing Huang, Peijie Wu

**Affiliations:** ^1^ Chengdu University of Traditional Chinese Medicine, Chengdu, China; ^2^ Zigong Hospital of Chengdu University of Traditional Chinese Medicine, Zigong, China

**Keywords:** tumor drug resistance, TME, SASP, redox dyshomeostasis, senescence, clinical therapy

## Abstract

The persistent challenge of tumor drug resistance remains a critical issue in medical practice, particularly during anti-neoplastic therapies, where the plasticity of the tumor microenvironment (TME) significantly complicates clinical treatment. Cellular senescence, an irreversible and permanent arrest of the cell cycle, has been implicated in various vital physiological and pathological processes. However, increasing evidence suggests that senescent cells arising in the tumor microenvironment have emerged as key contributors to tumor drug resistance, primarily through a highly active secretome termed the senescence-associated secretory phenotype (SASP), which includes growth factors, chemokines, cytokines, and stromal metalloproteinases. These SASP secretions significantly reshape the TME, enabling cancer cells to evade immune destruction. Interestingly, redox signaling networks are deeply intertwined with the cellular senescence process, influencing tumor progression and therapeutic outcomes. These studies highlight the complexity and heterogeneity of cellular senescence and redox signaling in diverse cancers. Notably, characterizing the heterogeneity of senescent cell populations in the context of drug resistance could facilitate the identification of key signaling nodes. Therefore, a thorough comprehension of the adaptive interactions between redox signaling and senescence across various tumor stages and cell subsets may reveal novel therapeutic targets. In this review, we will interpret the role of redox signaling in driving senescence and its regulation of SASP secretion in TME. Additionally, we will provide insights into existing and emerging clinical interventions that harness redox modulation to improve therapeutic efficacy while minimizing adverse effects. Together, co-targeting tumor cells and senescent counterparts in the tumor microenvironment may provide the potential to achieve enhanced therapeutic benefits and restrain tumor relapse in future clinical oncology.

## 1 Introduction

Tumor drug resistance remains a formidable obstacle in modern oncology, undermining the long-term efficacy of both conventional and targeted therapies. Although initial treatment responses are often clinically promising, resistance frequently develops, leading to recurrence, metastasis, and a poor prognosis (Nussinov et al., 2021). Over the past decade, increasing research has focused on the complexity and plasticity of tumor microenvironment (TME) underlying tumor drug resistance. Recently, senescent cells arising in the TME have emerged as a pivotal and underexplored axis that plays a key role in restraining therapy resistance.

Cellular senescence is regarded as a fundamental response of tumor cells to therapy. There is growing evidence that points to the accumulation of senescent cells in patients receiving radiation therapy or chemotherapy. For example, the term “therapy-induced senescence” (TIS) refers to a stress response induced by anticancer treatments that culminate in a stable cell cycle arrest. Cellular senescence itself is a durable form of growth arrest triggered by various stressors, including DNA damage, oncogene activation, and exposure to chemotherapeutic or radiotherapeutic agents (Di Micco et al., 2021). While traditionally viewed as a tumor-suppressive process, senescent tumor and stromal cells frequently acquire a SASP (Davalos et al., 2010). The SASP is characterized by the release of pro-inflammatory cytokines, growth factors, and proteolytic enzymes in the TME (Gabai et al., 2023; You and Wu, 2025). These factors can promote tumor progression, and therapeutic resistance. Interestingly, redox signaling, primarily mediated by reactive oxygen species (ROS) and tightly regulated by endogenous antioxidant systems, plays a central role in both initiating and sustaining senescence (Javali et al., 2023). On the one hand, elevated ROS levels cause oxidative DNA damage and mitochondrial dysfunction, which trigger senescence. On the other hand, persistent oxidative stress within senescent cells further amplifies SASP activity, creating a pro-tumorigenic microenvironment that fosters resistance (Palma et al., 2024; Yang et al., 2024). This redox-senescence feedback loop is increasingly recognized as both a driver and a consequence of tumor adaptation under therapeutic pressure. Therefore, understanding and harnessing the interplay between redox signaling and senescence may provide a novel and effective strategy to overcome tumor drug resistance. By targeting key molecular mediators and signaling pathways within this interaction, it may be possible to develop therapies that not only eliminate cancer cells but also suppress the emergence of resistant subpopulations (Yang et al., 2024; 2025). Additionally, we summarize current clinical approaches and emerging strategies that modulate the redox-senescence axis, providing a foundation for deeper exploration and innovation in this field. Notably, the application of nanomaterials to modulate redox dyshomeostasis has emerged as a promising Frontier in cancer therapy, providing new tools to selectively disrupt the redox balance in tumor cells. A comprehensive understanding of this interaction could ultimately guide the development of more durable, adaptive, and personalized cancer therapies.

The SASP, a hallmark of senescent cells, exerts dual roles in tumor progression and drug resistance. In early tumorigenesis, cellular senescence prevents malignant proliferation and mutation accumulation as a tumor-suppressive mechanism (Colucci et al., 2025). As tumors progress, however, SASP components exhibit pro-tumorigenic functions: growth factors stimulate tumor cell proliferation; TGF-β and IL-6 induce epithelial-mesenchymal transition to enhance invasion (Song et al., 2023). Concomitantly, VEGF promotes angiogenesis to fuel tumor growth, and immunosuppressive cell recruitment coupled with effector T-cell inhibition facilitates tumor evasion of immune surveillance (Mahaki et al., 2025). In the context of therapeutic resistance, radio/chemotherapy induces senescence in the TME. Resultant SASP factors induce phenotypic alterations in residual cancer cells, conferring multidrug resistance—e.g., SPINK1 from senescent stroma activates survival pathways in adjacent cancer cells (Gabai et al., 2023). Collectively, evidence positions SASP as a dynamic therapeutic target. Elucidating its mechanisms is critical for overcoming resistance and improving clinical outcomes.

## 2 Senescence in tumor drug resistance

### 2.1 Hallmarks of cell senescence in tumor drug resistance

**FIGURE 1:**
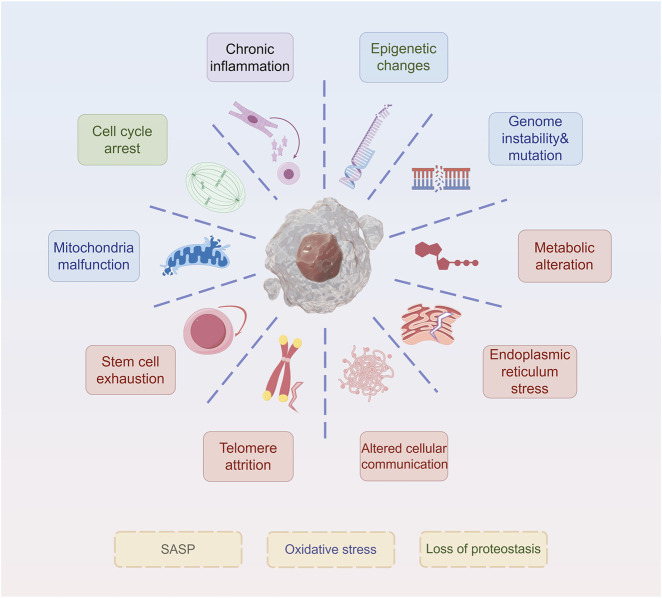
Hallmarks of senescence. The ten hallmarks of senescence include chronic inflammation, cell cycle arrest, mitochondria malfunction, stem cell exhaustion, telomere attrition, epigenetic changes, genome instability&mutation, metabolic alteration, endoplasmic reticulum stress, and altered cellular communication.

Similar to other types of senescence, such as replicative and oncogene-induced senescence, therapy-induced senescent (TIS) cells exhibit core morphological and molecular hallmarks, including an enlarged, flattened, and irregular morphology, increased lysosomal content, and depletion of Lamin B1 (Bajtai et al., 2025). Here, we discuss the current mainstream signs of aging (Figure 1). These characteristics are readily detectable and quantifiable through standard laboratory techniques, including microscopy and immunoblotting. Notably, senescence-associated β-galactosidase (SA-β-gal) activity, detectable histochemically at pH 6.0 (compared to its optimal pH of 4.0 in non-senescent cells), remains the most widely used biomarker. SA-β-gal-positive tumor cells persist after treatment and secrete pro-survival factors that protect neighboring cells, thereby contributing to therapeutic resistance (Mirzayans and Murray, 2023). Recent research highlights the influence of redox regulation on SA-β-gal activity. Increased reactive oxygen species (ROS) stabilize lysosomal membranes and enhance β-galactosidase retention, whereas antioxidant systems, such as glutathione (GSH), counteract this effect (Li et al., 2025). This redox-sensitive regulation suggests that SA-β-gal is not merely a biomarker but also a functional mediator of the SASP, which can promote drug tolerance (Bulbiankova et al., 2023). In addition, Lamin B1, a structural component of the nuclear lamina, is downregulated during senescence, leading to nuclear envelope destabilization and chromatin remodeling (Lv et al., 2024). In therapy-resistant tumors, the loss of Lamin B1 is associated with epigenetic reprogramming and the reactivation of pro-proliferative gene programs (Sadida et al., 2023). Oxidative stress accelerates Lamin B1 degradation through ROS-mediated post-translational modifications, further linking redox imbalance to changes in nuclear architecture (Kristiani and Kim, 2023). Targeting Lamin B1-deficient cells using senolytic agents, especially in combination with ROS-inducing compounds, has shown promise in eliminating resistant cell populations.

Persistent DNA damage is a defining feature of TIS cells, particularly in the early stages of senescence. DNA double-strand breaks (DSBs) activate ATM and ATR kinases, initiating a cascade that leads to phosphorylation of signaling proteins such as Nijmegen breakage syndrome (NBS1) and the histone variant H2AX (γH2AX), ultimately resulting in p53 activation and transactivation of CDK inhibitors (Láscarez-Lagunas et al., 2022). The severity of DNA damage can range from localized DSBs to genome-wide instability, including aneuploidy, chromosomal rearrangements, micronuclei formation, and the emergence of extrachromosomal DNA fragments (Sebastian et al., 2025). However, resistant tumors often exhibit an attenuated DNA damage response or upregulate repair mechanisms. For instance, cancers resistant to poly (ADP-ribose) polymerase (PARP) inhibitors enhance base excision repair (BER) pathways to bypass senescence (Vickridge et al., 2022). ROS exhibit dual roles in this context: they initiate DNA damage to trigger senescence yet also activate antioxidant transcription factors such as NRF2, which mitigate oxidative stress and attenuate the DDR (Ngo and Duennwald, 2022). This duality underscores the therapeutic need for redox-balanced combination strategies to reinforce DNA damage-induced senescence.

### 2.2 Heterogeneity of senescence in tumor drug resistance

Although senescence-associated biomarkers are well-defined, senescent cells (SCs) exhibit significant heterogeneity, particularly in their secretory phenotypes. This heterogeneity emerges during tumor development, wherein therapeutic interventions exert selective pressure on diverse SC populations, consequently driving the evolution of drug resistance (Ottaiano et al., 2023; Proietto et al., 2023; Tyler et al., 2025). The multifaceted role of SC heterogeneity in tumor resistance manifests primarily through three mechanisms:

First, SCs evade apoptosis by activating distinct anti-apoptotic pathways (Salminen et al., 2010; Liu et al., 2020; Qiang et al., 2025). A key mechanism by which specific cancer stem cell (CSC) subpopulations confer therapy resistance is their ability to evade programmed cell death, facilitated by persistent activation of anti-apoptotic signaling networks. The PI3K/AKT/mTOR axis is frequently hyperactivated in therapy-induced senescent cells, providing a critical survival signal that counteracts pro-apoptotic stimuli. Activation of this pathway suppresses pro-apoptotic proteins (e.g., BAD) while promoting anti-apoptotic BCL-2 family members (e.g., BCL-2, BCL-XL) (Karami Fath et al., 2022; Son et al., 2024). Concurrently, CSCs often exhibit dysregulation of the extrinsic apoptotic pathway. Although some stimuli induce apoptosis in CSCs (Wang X. et al., 2022), subsets develop resistance to death receptor-mediated apoptosis via upregulated cellular FLICE-inhibitory protein (c-FLIP) – blocking caspase-8 activation–or downregulated receptors (e.g., FAS/CD95) (Chu et al., 2024; Yaacoub et al., 2024). The cumulative effect of these anti-apoptotic adaptations endows CSCs with remarkable resilience against cytotoxic therapies designed to eradicate tumor cells. Second, certain SCs bypass growth arrest via elevated Cyclin-Dependent Kinase 1 (CDK1) expression and polyploidization (Basisty et al., 2020). Heterogeneity enables some SC subpopulations to escape senescence-associated cell cycle arrest, acquiring proliferative potential that fuels recurrence. Elevated CDK1 expression/activity drives this escape. Normally suppressed in senescence, CDK1 reactivates in SC subsets via suppression of CDK inhibitors (e.g., p21CIP1/WAF1) or oncogenic pathways (Wang et al., 2023), enabling aberrant cell cycle re-entry and circumvention of G2/M arrest (Fernando et al., 2021). Polyploidization (whole-genome doubling) in stress-exposed SCs generates polyploid giant cells. These undergo depolyploidization via asymmetric division, yielding diploid progeny with genomic alterations and stem-like properties that drive tumor heterogeneity and resistance (Li et al., 2022; Massacci et al., 2023). Additionally, therapy-induced senescent cells may acquire stem-like properties through phenotypic reprogramming, enhancing their resistance and invasiveness following senescence escape (Saleh et al., 2020). The senescence program itself, or the process of escaping it, can actively facilitate the acquisition of stem-like traits in specific SC subpopulations, thereby significantly contributing to tumor aggressiveness and therapy resistance. SCs, especially those that emerge following therapy or in specific tumor microenvironments, can upregulate core pluripotency factors including SOX2, OCT4, and NANOG (Swain et al., 2020; Chen and Wang, 2025; Hwang et al., 2025). This transcriptional reprogramming confers upon them enhanced self-renewal capacity, multipotency, and tumor-initiating potential. Activation of key stemness-related signaling pathways is critical. For example, the Wnt/β-catenin pathway, frequently reactivated in SCs that evade growth arrest, promotes stemness and cell survival (Wu et al., 2025). Similarly, the Notch and Hedgehog pathways are often involved in maintaining the stem-like state of senescent or senescence-evaded cells. These stem-like SCs are resistant to conventional therapies (which target dividing cells while sparing quiescent stem cells) and serve as reservoirs for tumor regeneration and metastasis (Kumar et al., 2021).

Senescent cells exhibit defined biomarkers but display significant heterogeneity, particularly in their secretory phenotype. Several studies have compared senescence induced by doxorubicin and CDK4/6 inhibitors (CDK4/6i), highlighting the heterogeneity of SASP responses (Wang B. et al., 2022). While both agents induce senescence, doxorubicin-induced SCs exhibit a pro-inflammatory, NF-κB-driven SASP, whereas CDK4/6i-induced SCs display a less inflammatory, p53-driven SASP, which supports immune surveillance. Interestingly, CDK4/6i treatment has been found to enhance anti-tumor immunity by suppressing regulatory T cells (Tregs) and promoting cytotoxic T-cell activity (Goel et al., 2017). The complexity and plasticity of SASP provide therapeutic opportunities. Modulating SASP composition through NF-κB inhibition has been shown to reduce tumor-promoting secretions, with metformin and mTOR inhibitors (e.g., rapamycin) effectively suppressing NF-κB-driven SASP(Liang et al., 2021; Wang L. et al., 2022; You and Wu, 2025). Additionally, inhibition of the p38MAPK pathway mitigates SASP-driven bone loss and metastasis in breast cancer models (Murali et al., 2018). Recent studies also suggest that epigenetic regulators such as KDM4 and EZH2 modulate SASP composition, influencing immune surveillance and tumor resistance (Zhang et al., 2021; Chibaya et al., 2023). While targeting SASP for anti-tumor therapy appears promising, caution is required due to the context-dependent effects of SASP modulation. For instance, in cyclophosphamide-induced senescence in lymphoma models, NF-κB inhibition paradoxically shortened overall survival by impairing SASP-mediated immune responses (Wang B. et al., 2024). Similarly, SASP modulation in CDK4/6i-induced senescence may not always be beneficial, as its p53-driven secretory response enhances anti-tumor immunity (Wang B. et al., 2022). Therefore, understanding how the various stimuli and activations of different aging procedures affect the SASP phenotype is the key to formulating a new type of SASP programming and improving intervention measures.

Overall, senescence heterogeneity is driven by dynamic alterations in the tumor microenvironment, genetic mutations, epigenetic modifications, and plasticity in signaling pathways (Wang Z. et al., 2024). Notably, the senescence heterogeneity in TME provides an additional mechanism by which tumor cells rapidly adapt to therapeutic pressures, contributing to genetic heterogeneity and therapeutic resistance (Balázsi et al., 2011; Turner et al., 2017; Bailey et al., 2020; Wu et al., 2022a). Understanding these diverse senescence phenotypes and their implications for drug resistance is crucial for designing effective senescence-targeted therapies.

## 3 Redox dyshomeostasis and senescence in tumor drug resistance

### 3.1 Redox dyshomeostasis induced persistent DNA damage and repair-driven senescence in tumor drug resistance

**FIGURE 2:**
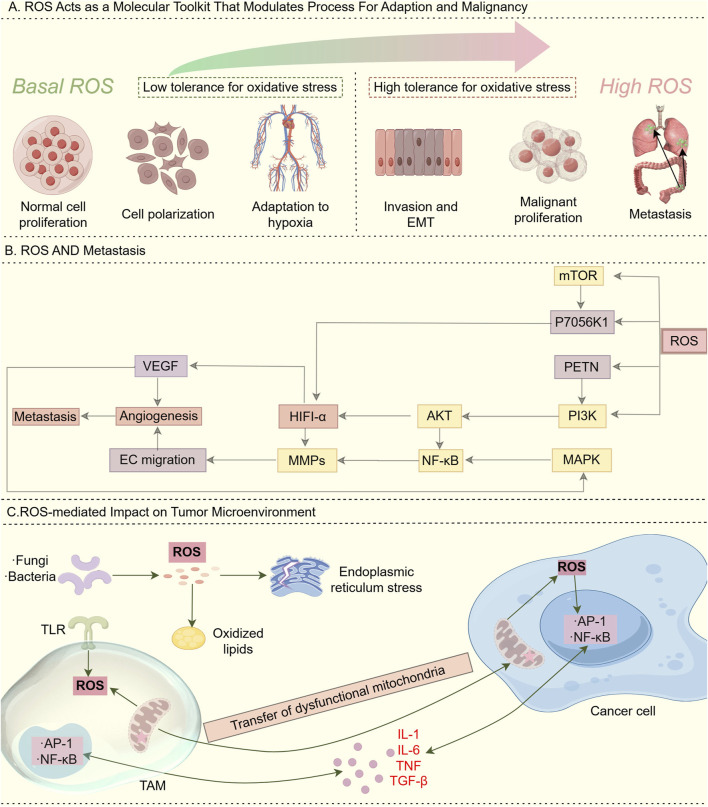
Role of ROS. **(A)** ROS acts as a “molecular toolkit” that modulates processes for adaptation and malignancy: Basal (low) ROS levels: In normal physiological environments, ROS/redox signaling acts as a physiological rheostat to regulate critical cellular functions for development and adaptation to the surrounding milieu. This maintenance of redox homeostasis is essential to normal cellular activity. High ROS levels: During cancer initiation, intrinsic factors such as oncogenic lesions, and external factors such as the chronic inflammatory environment, shift redox homeostasis into a “pro-oxidant” state. This “pro-oxidant” state in turn facilitates pro-tumorigenic behaviors and selects for higher tolerance against oxidative stress. In this way, alterations in ROS and redox signaling allow fine-tuned adjustments that underlie both physiological and pathological conditions. **(B)** ROS and metastasis. ROS production stimulates the induction of PI3K/AKT/mTOR and MAPK pathways that trigger metastasis. **(C)** ROS-mediated Impact on tumor microenvironment: ROS generated in the tumor microenvironment through various mechanisms, including aberrant metabolism due to dysfunctional mitochondria, toll-like receptor (TLR) activation, and intratumoural microorganisms, can stimulate redox-sensitive pathways such as NF-κB and AP-1 in cancer cells and tumor-associated macrophages (TAMs), resulting in the release of inflammatory cytokines and chronic inflammation.

Redox homeostasis serves as an intrinsic defense mechanism essential for maintaining cellular physiological stability (Li et al., 2025). The oxidative stress response system and its associated signaling pathways are susceptible to fluctuations in the redox environment (Chang et al., 2025), ensuring a tightly regulated balance between reactive oxygen species (ROS) generation and clearance (Lu et al., 2023). ROS and redox signaling alterations enable fine-tuned regulation underlying both physiological adaptation and pathological malignancy (Li et al., 2025), with basal ROS acting as a physiological rheostat and high ROS promoting tumorigenesis via pro-oxidant state induction (Figure 2A). Additionally, ROS production triggers metastasis by stimulating the PI3K/AKT/mTOR and MAPK signaling pathways (Caligiuri et al., 2023) (Figure 2B). In terms of effects of ROS on immunity. Tumor microenvironment-derived ROS, generated through mitochondrial dysfunction, TLR activation, or intratumoral microorganisms, activates redox-sensitive NF-κB/AP-1 pathways in cancer cells and TAMs, leading to chronic inflammation via inflammatory cytokine release (Glorieux et al., 2024) (Figure 2C). Excessive ROS triggers the antioxidant defense (AOD) system, leading to metabolic exhaustion in mitochondria, peroxisomes, and the endoplasmic reticulum, ultimately causing severe redox imbalance (Lennicke and Cochemé, 2021; Wei et al., 2025).

Dysregulated redox homeostasis is closely linked to tumor initiation and progression, primarily through oxidative modifications that induce DNA damage response (DDR) (Wang Y. et al., 2021). ROS-mediated oxidative DNA lesions, such as 8-hydroxy-2′-deoxyguanosine (8-OHdG), are among the most abundant oxidative DNA damage markers and are significantly elevated in various cancers (Zhao et al., 2023). The loss of tumor suppressor genes such as p53, ATM, and FoxO3 correlates with increased ROS levels, exacerbating genomic instability (Lu et al., 2015; Hartwig et al., 2021). Notably, p53 functions as a critical guardian of the genome, preventing oxidative damage, but is often downregulated in ROS-induced cancer cells (Lee, 2024). Similarly, ATM acts as a mediator of DDR and oxidative stress response, while FoxO3 regulates ROS homeostasis through ATM signaling (Zhu et al., 2024). The Nrf2-Keap1 pathway plays a pivotal role in mitigating redox imbalances by inducing antioxidant enzymes such as GST, GPx, and superoxide dismutases (Sun Z. et al., 2025). However, in cancer, mutations in Keap1 or activation of oncogenic RAS disrupt this balance, leading to elevated mitochondrial biogenesis, increased ROS levels, and enhanced DDR (Punekar et al., 2022). Oncogenic RAS mutations also promote ROS accumulation through Nox4-p22phox activation, with NOX4 identified as a key mediator of RAS-driven DDR (Ford et al., 2020). Studies indicate that K-Ras activation significantly increases DNA strand breaks and peroxide levels, further amplifying genomic instability (Igarashi et al., 2023).

While ROS-induced DDR contributes to tumorigenesis, it also drives tumor resistance mechanisms (Chauhan et al., 2024; Ma et al., 2025). First, DDR facilitates genomic mutations, enabling tumors to evade therapy (Drew et al., 2025). For example, aberrant DDR activation has been linked to resistance in acute myeloid leukemia (AML) and chronic myeloid leukemia (CML), with FLT3-ITD and Bcr-Abl mutations conferring resistance to tyrosine kinase inhibitors (TKIs) such as imatinib (Huang and Zhou, 2021). Second, tumors enhance DNA repair capacity, mitigating the cytotoxic effects of DNA-damaging agents. Homologous recombination (HR) and non-homologous end joining (NHEJ) are two primary pathways for repairing DNA double-strand breaks (DSBs) (Huang and Zhou, 2021; Li Q. et al., 2023). Notably, ATM signaling plays a critical role in coordinating DDR by phosphorylating key substrates involved in DNA repair, checkpoint activation, and apoptosis (Lee, 2024). Recent studies suggest that extrachromosomal DNA (ecDNA) formation represents an additional DDR-driven mechanism of tumor resistance. If simultaneous chromosomal DNA breaks occur, DNA fragments may undergo rearrangement, leading to the formation of small circular DNA structures (ecDNA). These ecDNA elements amplify oncogenes, enhance tumor heterogeneity, and confer resistance to prior treatments (Koche et al., 2020; Bailey et al., 2024). For instance, RAB3B ecDNA amplification induces cisplatin resistance via autophagy activation in hypopharyngeal squamous cell carcinoma (HSCC) (Lin et al., 2022). Similarly, in lung cancer and melanoma models, tumors harboring BRAF^V600E ecDNA amplification exhibit resistance to ERK inhibitors (Lin et al., 2022). Given its role in tumor adaptation and resistance, ecDNA holds promise as a biomarker for cancer diagnosis, prognosis, and prediction of treatment response. Further investigation into DDR-driven ecDNA generation may provide new avenues for targeted cancer therapies.

### 3.2 Oxidative stress mediates senescence-associated secretory phenotype in tumor drug resistance

Cellular senescence is widely recognized as a protective mechanism that suppresses tumorigenesis by inducing cell cycle arrest, inhibiting proliferation, and preventing oncogenic mutations (Schmitt et al., 2022). Consequently, anticancer therapies frequently induce senescence to halt tumor progression (Liu B. et al., 2025). While this approach effectively halts cancer cell proliferation, senescent cells remain metabolically active and can exert unintended effects, primarily through the senescence-associated secretory phenotype (SASP) (Milanovic et al., 2018). Persistent cellular stressors, including oxidative stress and the DNA damage response (DDR), can trigger the SASP in both malignant and non-malignant cells within the TME (Wang et al., 2020; Wu et al., 2021). SASP contributes to epithelial-to-mesenchymal transition (EMT), local tissue invasion, angiogenesis, fibroblast activation, immunosuppression, metastasis, and therapy resistance (Birch and Gil, 2020; Nacarelli et al., 2020; Ruscetti et al., 2020; Dong Z. et al., 2024; Yamashita and Kumamoto, 2024). However, SASP also enhances chemotherapy-induced immune clearance by recruiting natural killer (NK) cells, macrophages, and cytotoxic T cells, thereby facilitating the removal of senescent cancer cells and potentially impeding tumor progression (Coppé et al., 2008; Ruscetti et al., 2020; Chibaya et al., 2022). The dual role of SASP—both tumor-promoting and tumor-suppressive is highly context-dependent, influenced by the source of cellular stress and the specific cell types involved (Basisty et al., 2020).

Chemotherapy, a conventional cancer treatment strategy, induces DNA damage response (DDR) via oxidative stress, thereby triggering SASP in both cancerous and surrounding stromal cells within the TME (Table 1). For instance, paclitaxel, a standard chemotherapeutic agent for triple-negative breast cancer (TNBC), induces endoplasmic reticulum (ER) stress by increasing inositol-requiring enzyme (IRE1α) RNase activity (Robinson et al., 2025). Elevated IRE1α activity enhances the expression of SASP-related factors, including IL-6, IL-8, CXCL1, granulocyte-macrophage colony-stimulating factor (GM-CSF), and TGF-β2 (Yang et al., 2021). Similarly, etoposide induces senescence in non-malignant cells, including smooth muscle cells, dermal fibroblasts, and other stromal components (Gao et al., 2023). Multiple myeloma (MM) cells exposed to doxorubicin exhibit reduced CD138 expression and increased CD45/CD20 levels, hallmarks of MM stemness, correlating with the upregulation of SASP factors, particularly RANTES. This phenomenon facilitates tumor cell re-entry into the cell cycle, thereby promoting therapy resistance (Riccardi et al., 2024). Additionally, ascorbic acid-induced senescence in breast cancer cells results in the secretion of eosinophil-associated factors, CXCL5, and RANTES, enhancing tumor cell proliferation (Choi et al., 2020). In malignant pleural mesothelioma, senescent cells induced by pemetrexed exhibit a pro-EMT SASP profile, promoting tumor-initiation potential (Tsao et al., 2022). Similarly, fibroblast-derived matrix metalloproteinases (MMPs) induced by bleomycin treatment have been shown to stimulate MDA-MB-231 breast cancer cells in xenograft models (Ju et al., 2024). Furthermore, IL-6 secretion induced by platinum-based chemotherapy promotes cancer stem cell (CSC) enrichment in high-grade serous carcinoma (HGSC), facilitating CSC expansion from dormant senescent cells, a crucial driver of tumor recurrence (Wang et al., 2018). Recent studies by Nacarelli et al. demonstrated that inhibition of nicotinamide phosphoribosyltransferase (NAMPT), a key SASP regulator, using FK866 suppresses platinum-induced CSC expansion (Nacarelli et al., 2020). Given the complexity and heterogeneity of SASP, its precise role in tumor progression remains challenging to delineate. However, further elucidation of context-dependent SASP functions may provide novel insights for tumor-targeted therapeutic strategies. Understanding the molecular determinants of SASP heterogeneity holds the potential to refine senescence-targeting therapies, enhance the efficacy of cancer treatments, and mitigate therapy resistance.

**TABLE 1:** Chemotherapy triggering SASP in cancer cells and surrounding cells in TME.

PMID	Senescence inducer	Type of cancer	Senescent cells	SASP factor	Major role of the SASP
30111846	Paclitaxel	Triple-negative breast cancer (TNBC)	ECs, mononuclear cells in the bone marrow, and fibroblasts	IL-6, IL-8, CXCL1, granulocyte-macrophage colony-stimulating factor (GM-CSF) and TGFβ2	Expansion of tumor-initiating cells
30518684	Platinum treatment	High-grade serous carcinoma (HGSC)	Fibroblasts	IL-6	OCSC enrichment in residual tumors、the expansion of CSCs from dormant senescent cells
23254289	Doxorubicin	Multiple myeloma (MM)	Smooth muscle cells, dermal fibroblasts, and other stromal cells	Rantes	Tumor cells evade senescence by re-entering the cell cycle, acquiring tumor-initiating capabilities
34985934	Pemetrexed	Malignant pleural mesothelioma (MPM)	Malignant pleural mesothelioma cells	ALDH (aldehyde dehydrogenase)	Amplify EMT-like clonogenic capacities and elevate levels of tumor-initiating capabilities
25263564	Docetaxle	PCa GEMM	Stromal call	SA-βgal, p16, p21, GM-CSF, CSF-1, IL-10, CCL-2, CXCL1/2	Impair tumor response
37017266	Platinum	Esophageal squamous cell carcinoma (ESCC)	ESCC cells	IL-1α, IL-1β, IL-6, IL-8	Enhance migration and invasion abilities
17409418	Bleomycin	Breast cancer	Fibroblasts	Matrix metalloproteases	Increase the proliferative capacity
22698404	Doxorubicin	Breast cancer	Mammary epithelial cell	Eotaxin, CXCL5, Rantes	Mitogenic support
25156255	Docetaxle	Prostate tumors	Prostate epithelial calls	MDSCs	Tumor promotion
35674109	Chemotherapy-induced DDR	Breast cancer	Cancer cells	Proinflammatory cytokine	SASP promotes angiogenesis and epithelial-mesenchymal transition
40119436	Cisplatin	Ovarian cancer	Cancer cells, Endothelial cells	MMP-3, PAI-1	Angiogenesis, stromal remodeling
39034318	Paclitaxel	Lung cancer	Cancer cells, Fibroblasts	IL-1β, TNF-α	Pro-inflammatory signaling, therapy resistance
30123112	Etoposide	Glioblastoma	Cancer cells, Microglia	TIMP-1, GRO-α	Immune cell recruitment, invasion
26042506	Docetaxel	Prostate cancer	Cancer cells, Fibroblasts	EGF2, PDGF-AA	Stromal activation, tumor growth
40011944	Temozolomide	Glioblastoma	Cancer cells, Astrocytes	SerpinE1, LIF	Pro-survival signaling, invasion
31631444	Bleomycin	Lung cancer	Epithelial cells, Fibroblasts	TGF-β, CTGF	Fibrosis, EMT
36717670	Carboplatin	Ovarian cancer	Cancer cells, Mesothelial cells	1L-11, SDF-1	Peritoneal dissemination
39034318	lrinotecan	Colorectal cancer	Cancer cells, Dendritic cells	CCL-5, GM-CSF	Immune cell activation, antigen presentation
38956429	Methotrexate	Osteosarcoma	Cancer cells, Osteoblasts	BMP-2, OPG	Bone remodeling, differentiation
31130723	Sorafenib	Hepatocellular	Cancer cells, Hpaticstellate cells	HGF, PDGF-BB	Fibrosis, angiogenesis
22544021	Oxalipla-tion	Colorectal cancer	Cancer cells, Macrophages	IL-6, IL-8, TGF-β	Promotes tumor recurrence, immune evasion

### 3.3 ROS and oxidative stress effect on senescence-associated immune microenvironment

**FIGURE 3:**
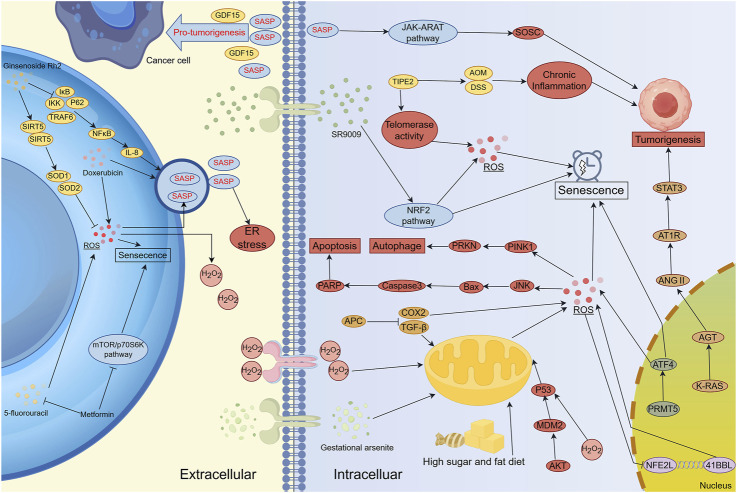
The Regulatory Network of Oxidative Stress and Senescence in Carcinogenesis, Cancer Progression and Therapy Resistance. Lifestyle factors, pharmacological agents, and targeted regulation of genes and signaling pathways (e.g., NRF2 and STAT3) can induce oxidative stress-mediated cellular senescence. However, the SASP secreted by senescent cells can paradoxically promote tumor development and progression. Several clinical treatment strategies combine conventional therapies with novel agents to mitigate oxidative stress-induced cellular senescence, thereby reducing resistance and associated adverse effects. SASP serves as a major communication route between senescent cells and both cancer and stromal cells, influencing immune modulation, extracellular matrix remodeling, and angiogenesis. Inhibition of tumor progression can be achieved by targeting senescence-inducing genes or signaling pathways involved in oxidative stress regulation. Notably, cancer-associated fibroblasts (CAFs) secrete SASP factors that facilitate tumor progression. Interestingly, cancer cells can also elevate ROS production in CAFs, reinforcing the pro-tumorigenic microenvironment and establishing a feed-forward loop that sustains malignancy.

Oxidative stress-induced senescence occurs in various cell types throughout the body, with the immune system serving as a key regulatory network (Figure 3). Oxidative stress within immune cells can lead to systemic dysfunction, affecting both innate and adaptive immune responses (Nakamura and Takada, 2021). At the cellular level, oxidative stress-induced senescence increases the adhesion of neutrophils and leukocytes to endothelial cells, impairing their migration to infection sites and diminishing chemotactic responsiveness (Scioli et al., 2020). This alteration stimulates the production of integrins and mucins, further enhancing adhesion to endothelial cells and impeding the migration of immune cells (Scioli et al., 2020). When cells are exposed to excessive ROS, they can enter a SASP-driven senescent state to prevent malignant transformation. Additionally, SASP can recruit immune cells to sites of tissue damage or enhance immune responses (Yasuda and Alan Wang, 2025). For example, CCL2 secreted by senescent liver cells has been shown to promote the recruitment of bone marrow-derived macrophages, contributing to inflammatory microenvironments (Liu et al., 2024). Furthermore, the genetic loss of the CCL2 receptor reduces macrophage infiltration in mice and increases the risk of hepatocellular carcinoma (Dong Y. et al., 2024). Several therapeutic strategies have been explored to manipulate oxidative stress-related senescence in cancer therapy. For instance, metformin treatment enhances the expression of NKG2D ligands (RAE-1 and MULT-1) and DNAM-1 ligands (PVR/CD155) on multiple myeloma (MM) cells, thereby increasing NK cell recognition and cytotoxicity against tumor cells (Molfetta et al., 2019). Similarly, in murine ovarian cancer models, cisplatin treatment sensitizes tumors to PD-1 blockade, a response correlated with dendritic cell (DC) and CD8^+^ T cell activation within the tumor microenvironment (Hao et al., 2021).

However, SASP induced by ROS and oxidative stress exhibits dual roles in cancer progression. While it recruits macrophages and NK cells for immune clearance, it can paradoxically facilitate tumor progression (van Weverwijk and de Visser, 2023). In a pancreatic cancer mouse model, the genetic ablation of Sin3B, a chromatin scaffold protein essential for senescence induction, resulted in delayed tumor growth, an effect associated with reduced expression of SASP factors, including IL-1 and IL-6 (Wang B. et al., 2024). Moreover, MEK and CDK4/6 inhibitors have been found to induce senescence in fibroblasts, triggering the secretion of neutrophil-derived (ND) factors that recruit myeloid-derived suppressor cells (MDSCs) into the TME, ultimately suppressing antitumor immunity (Crozier et al., 2023). Additionally, ROS can regulate T-cell proliferation and activation (Morris et al., 2022). Chronic exposure to elevated reactive oxygen species (ROS) levels induces DNA damage response (DDR), potentially leading to the formation of extrachromosomal DNA (ecDNA). Interestingly, ecDNA harboring immunomodulatory genes has been linked to decreased tumor-infiltrating T cell populations, which contributes to immune evasion and dysfunctional T cell responses (Bailey et al., 2024). Excessive ROS exposure can also inhibit T cell proliferation and induce T cell apoptosis, further compromising immune surveillance (Zheng et al., 2023).

## 4 Harnessing the interaction between redox and senescence in tumor therapy

Cellular redox homeostasis refers to the dynamic balance between oxidants and antioxidants, which is crucial for regulating physiological processes, including cell proliferation, metabolism, differentiation, senescence, and apoptosis (Li et al., 2025). Oxidants are chemical species that acquire electrons during oxidation reactions, including reactive oxygen species (ROS) and reactive nitrogen species (RNS). Moderate levels of oxidants are essential for cellular signaling, biosynthesis, and host defense (Ghoneum et al., 2020; Sharma et al., 2023). Under conditions of chronic oxidative stress, the antioxidant defense system is activated to mitigate ROS-induced cellular damage, highlighting the importance of maintaining redox homeostasis for cellular survival and growth (Afzal et al., 2023).

Compared to normal cells, cancer cells often exhibit elevated levels of oxidative stress due to genetic instability, chronic inflammation, and metabolic dysregulation (Selvaraj et al., 2025). These factors further drive gene mutations, inflammatory signaling, and ROS accumulation, forming a self-perpetuating cycle of oxidative stress, which creates a vicious circle and causes continuous oxidative stress. Cancer cells that survive under such stressful conditions develop adaptive mechanisms to enhance antioxidant defense systems, promoting resistance to ROS-mediated anticancer therapies, including chemotherapy, radiotherapy, photodynamic therapy (PDT), and chemodynamic therapy (CDT) (Hyun, 2020). Therefore, exploiting the interplay between redox signaling and senescence represents a promising therapeutic strategy to enhance tumor sensitivity to existing treatments.

### 4.1 Redox dyshomeostasis and senescence in current clinical therapy

#### 4.1.1 Strategies to target ROS in cancer

Recent evidence suggests that targeting ROS in cancer therapy can effectively eliminate malignant cells, induce minimal toxicity in normal cells, and overcome drug resistance. Based on their mechanisms of action, ROS-targeting strategies can be broadly classified into two categories: (1) strategies targeting ROS generation mechanisms and (2) strategies modulating redox adaptation pathways (Glorieux et al., 2024).

Strategies targeting ROS generation involve inhibiting NADPH oxidase (NOX) and nitric oxide synthases (NOS). NOX is overexpressed in multiple cancers, leading to excessive ROS production. Inhibiting NOX can reduce ROS levels, thereby suppressing tumor growth. For example, the NOX inhibitor GKT831, which targets CD44, has demonstrated efficacy in preclinical cancer models (Zhu et al., 2022; Pang et al., 2023). Similarly, NOS inhibitors have been shown to suppress the production of inflammatory cytokines and tumor invasion in cancer-associated fibroblasts (CAFs) (Xu et al., 2025). Conversely, strategies to enhance cytotoxic ROS levels focus on increasing ROS production to trigger oxidative stress-induced cancer cell death. For instance, upregulation of NOX5 via small-molecule agents (e.g., Anlotinib) promotes superoxide (O_2_·^-^) accumulation and apoptosis in synovial sarcoma cells (Huang et al., 2021). Additionally, pharmacological doses of pro-oxidant compounds, such as vitamin C, can amplify the Fenton reaction, making cancer cells more susceptible to radiotherapy and chemotherapy (Furqan et al., 2022; Petronek et al., 2024). Strategies targeting redox adaptation mechanisms primarily involve inhibiting endogenous antioxidants or disrupting upstream redox regulators. For example, blocking glutathione (GSH) synthesis leads to ROS accumulation in cancer cells, selectively inducing cancer cell apoptosis (An et al., 2024; Zhang et al., 2025). Phenethyl isothiocyanate (PEITC), a natural compound found in cruciferous vegetables, increases intracellular ROS levels and promotes cell death by inhibiting GSH synthesis (Zhen et al., 2023; Dong X. et al., 2024). Similarly, inhibitors of glutathione peroxidase (GPX) and thioredoxin reductase (TXNRD) shift cellular redox balance toward oxidative stress, triggering cancer cell death (Sun Y. et al., 2021; Tan et al., 2022; Augello et al., 2023; Liu et al., 2023; Peng et al., 2023). Beyond conventional redox-targeting approaches, light-based therapies, such as photodynamic therapy (PDT) and photothermal therapy (PTT), can induce cancer cell apoptosis by generating ROS and ER stress (Hou Y. et al., 2020). Additionally, some chemotherapeutic agents, such as cisplatin, induce ROS-mediated cytotoxicity, contributing to their anticancer effects (Zhang et al., 2022). ROS also plays a pivotal role in cancer stem cell (CSC) regulation, influencing stemness, survival, and therapy resistance. Targeting ROS in CSCs represents a promising strategy for eliminating therapy-resistant tumor populations. (Khan et al., 2023; Toledo et al., 2023). For example, PEITC and its synthetic analog LBL21 have been shown to increase ROS levels and deplete GSH in CSCs, reducing tumor formation capacity (Liu et al., 2022).

ROS-based therapeutic strategies offer a promising approach to cancer treatment, selectively targeting tumor cells while sparing normal tissues. The success of these strategies depends on a comprehensive understanding of redox dynamics in tumor cells and the development of advanced analytical techniques to monitor ROS species (Murphy et al., 2022). Numerous studies have highlighted the cytotoxic effects of ROS inducers on cancer cells by promoting oxidative cell death. ROS-inducing agents are classified by their primary cell death mechanisms: ferroptosis (Table 2) and apoptosis/alternative pathways (Table 3). This division reflects distinct redox vulnerability profiles in therapy-resistant tumors. As research continues to elucidate the complex role of ROS in tumor progression and therapy resistance, these strategies are expected to play an increasingly important role in future cancer treatments.

**TABLE 2:** ROS inducers for cancer therapy. Ferroptosis-inducing agents for cancer therapy.

PMID	Compound	Target	Type of cancers	Type of cell death	Stage of application
33827043	SGNI	HO-1	Tiple-negative breast cancer	Ferroptosis	Approved anti-cancer drug
38736312	Quercetin	mTOR	Oral squamous cell carcinoma	Ferroptosis	Preclinical
39332284	Daphnetin	NOQ1	Ovarian cancer	Ferroptosis	Preclinical
35041678	Curcumin	SLC1A5	Breast cancer	Ferroptosis	Phase Ⅱ(NCT00745143)
39664583	Trifluoperazine	SCL7A11	Oral cancer	Ferroptosis	Approved antipsychotic drug
39012393	Triptonide	SCL7A11	Colorectal cancer	Ferroptosis	Preclinical
39134409	Hesperadin	SCL7A11	Chronic myeloid leukemia	Ferroptosis	Preclinical
39393329	Gingerenone	SCL7A11	Colorectal cancer	Ferroptosis	Approved anti-cancer drug
32776119	Tan ⅡA	SCL7A11	Gastric cancer	Ferroptosis	Approved anti-cancer drug
34162423	Metformin	SCL7A11	Breast cancer	Ferroptosis	Approved anti-cancer drug
31043744	IFGN	SLC7A11	Fibrosarcoma, ovarian cancer	Ferroptosis	Approved immunomodulatory drug
24844246	Sorafenib	SLC7A11	Liver cancer	Ferroptosis	Approved anti-cancer drug
31616161	Sulfasalazine	SLC7A11	Pancreatic cancer, lung cancer	Ferroptosis	Approved antibiotics
22632970	Erastin	SLC7A11	Fibrosarcoma, lung cancer	Ferroptosis	Preclinical
32531676	Solasonine	GPX4	Liver cancer	Ferroptosis	Preclinical
37268198	Shokonin	GPX4	Non-small cell lung cancer	Ferroptosis	Preclinical
36396150	Trabectedin	GPX4	Non-small cell lung cancer	Ferroptosis	Approved anti-cancer drug
38126996	Acetylshikon-in	GPX4	Lung cancer	Apoptosis	Preclinical
37910602	N6F11	GPX4	Pancreatic cancer	Ferroptosis	Preclinical
34716292	FIN56	GPX4	Bladder cancer	Ferroptosis	Preclinical
30524291	RSL3	GPX4	Lung cancer, colorectal cancer	Ferroptosis	Preclinical
22297109	ML162	GPX4	Fibrosarcoma	Ferroptosis	Preclinical
22297109	ML210	GPX4	Fibrosarcoma	Ferroptosis	Preclinical
32596160	PdPT	GPX4	Lung cancer	Ferroptosis and apoptosis	Preclinical
31086302	Ferroptocide	TXN	Lung cancer, colorectal cancer	Ferroptosis	Preclinical
24439385	Buthionine sulfoximine	GCLC	Ovarian cancer, breast cancer	Ferroptosis and apoptosis	Phase Ⅰ(NCT00002730)
36814470	HO-3867	GPX4	Non-small cell lung cancer	Ferroptosis and apoptosis	Preclinical

**TABLE 3:** ROS inducers for cancer therapy. ROS-inducing apoptotic agents for cancer therapy (Focus on apoptosis/paraptosis/pyrotosis).

PMID	Compound	Target	Type of cancers	Type of cell death	Stage of application
31250512	L-mimosine	Tyrsinase	Melanoma	Apoptosis	Approved anti-cancer drug
33389727	Papaverine	Bce-Abl	Chronic myeloid leukemia	Apoptosis	Approved anti-cancer drug
32231343	Plumbagin	GPX4	Liver cancer	Apoptosis	Preclinical
26550453, 24172913	PX-12	TXN	Lung cancer, liver cancer	Apoptosis	Phase Ⅱ(NCT00417287)
12049479	2-Methoxyestradiol	SOD-1	Prostate cancer, leukemia	Apoptosis	Phase Ⅱ(NCT00502579)
18253124	ANT-224	SOD-1	Epodermoid carinoma	Apoptosis	Phase Ⅱ(NCT00405574)
35526323	Agronolide	SOD-1	Ovarian cancer	Apoptosis and ferroptosis	Preclinical
33488943	Herteronemin	SOD-2, MAPK	Hepatocellular carcinoma	Apoptosis	Preclinical
31715238	AGG	SOD-1, NRF-2	Oral cancer	Apoptosis	Preclinical
17572693	PMX464	TXN	Colorectal cancer	Apoptosis	Preclinical
33435380	Libertelle-none H	TXN	Pancreatic cancer	Apoptosis	Preclinical
37836684	Benzophenanthridine	TXNRD	Gastric cancer	Apoptosis	Preclinical
35569514	Diffractaic acid	TXNRD	Breast cancer	Apoptosis	Preclinical
34973363	Shikonin	TXNRD	Breast cancer	Apoptosis	Preclinical
31680962	Piperlongu-mine	TXNRD	Liver cancer	Apoptosis	Preclinical
36780763	Thimerosal	TXNRD	Lung cancer	Apoptosis	Preclinical
31176737	WZ26	TXNRD	Colon cancer	Apoptosis	Preclinical
38909408	Auranofin	TXNRD	Lymphoma	Apoptosis	Approved anti-rheumatoid arthritis drug
34850406	Auranofin	TXNRD	Lung cancer	Apoptosis	Approved anti-rheumatoid arthritis drug
30590310	B63	TXNRD	Gastric cancer	Paraptosis	Preclinical
33836250	Jolkinolide B	TXNRD	Bladder cancer	Paraptosis	Preclinical
31956937	Liquiritin	ROS	Hypatocellular carcinoma	Apoptosis	Preclinical
31407251	CBZ	ROS	Colon cancer	Apoptosis	Approved anti-cancer drug
36290582	Cisplatin	ROS	Esophageal adenocarcinoma	Apoptosis	Approved anti-cancer drug
25932150	Arsenic trioxide	ROS	Leukemia, myeloma	Apoptosis	Approved anti-cancer drug
12821677	Bortezomid	ROS	Lung cancer, multiple myeloma,	Apoptosis	Approved anti-cancer drug
25769405	Disulfiram	ROS	Breast cancer	Apoptosis	Approved anti-alcoholism drug
23837948	5-Fluorouracil	ROS	Colorectal cancer	Apoptosis	Approved anti-cancer drug
25937300	Nelfinavir	ROS	Cervical cancer	Apoptosis	Approved anti-HIV drug
22275514	Imexon	ROS	Pancreatic cancer	Apoptosis	Phase II (NCT00637247)
28033383	Withaferin	ROS	Breast cancer	Paraptosis	Phase II (NCT05610735)
21555567	Lanperisone	ROS	Lung cancer	Non-apoptosis	Approved muscle relaxant
36914635	Sanguinarine	ROS	Colorectal cancer	Oxeiptosis	Preclinical
34872005	Auriculasin	ROS	Colorectal cancer	Apoptosis, ferroptosis and oxeiptosis	Preclinical
37196512	Neobavaisof-lavone	ROS	Liver cancer	Pyroptosis	Preclinical

#### 4.1.2 Current therapeutic implications of oxidative stress and senescence in cancer treatment

The induction of cellular senescence is a frequent consequence of anticancer therapies, including chemotherapy and radiotherapy, and plays a significant yet complex role in treatment outcome (Oyama et al., 2024). Oxidative stress is a key driver of this therapy-induced senescence (Yang et al., 2022; Li D. et al., 2023). While senescence can act as a tumor-suppressive barrier, the persistence of senescent cells, particularly therapy-resistant senescent cancer cells and senescence in stromal cells, contributes substantially to adverse effects. These include the development of chemoresistance, tumor relapse and progression (often facilitated by the SASP) (Dong Z. et al., 2024; Ding et al., 2025), radiation resistance (Peng et al., 2020), and debilitating off-target toxicities such as cardiotoxicity and gastrointestinal damage (Demaria et al., 2016). This highlights the dual role of senescence in cancer therapy–a desired initial response can evolve into a driver of resistance and toxicity. Consequently, strategies targeting senescent cells have gained significant interest. Broadly, these interventions fall into two categories: senolytics, which selectively eliminate senescent cells (discussed in Section 4.1.3), and senomorphics, which modulate the detrimental functions of senescent cells, particularly the SASP (discussed in Section 4.1.4). Understanding and harnessing these distinct approaches offer promising avenues to overcome therapy resistance and mitigate treatment-related side effects.

#### 4.1.3 Senolytics for anticancer therapy

Senolytic therapy, a treatment strategy targeting senescent cells, has emerged as a promising approach in cancer therapy. The fundamental principle of this therapy is to eliminate therapy-induced senescent cells, thereby mitigating age-related cancer symptoms and promoting healthy aging. In oncology, senolytic therapy primarily aims to eliminate therapy-resistant senescent cancer cells that evade conventional chemotherapy and radiotherapy. These persistent senescent cells contribute to tumor progression, chronic inflammation, and therapeutic resistance.

The mechanism of senolytic therapy relies on the unique vulnerabilities of senescent cells. Senescent cells, particularly those induced by therapy, exhibit distinct molecular and metabolic signatures that pharmacological agents can selectively target. For instance, senescent cancer cells frequently upregulate anti-apoptotic BCL-2 family proteins, making them vulnerable to BCL-2 inhibitors (Vogler et al., 2025). Navitoclax (ABT-263), a potent inhibitor of BCL-2, BCL-XL, and BCL-W, has demonstrated efficacy in eliminating a broad range of senescent cells, including therapy-induced senescent cancer cells (Czajkowski et al., 2025). Navitoclax has also shown promise in clinical trials. When combined with rituximab, it exhibited superior efficacy compared to monotherapy in patients with chronic lymphocytic leukemia (CLL), leading to prolonged progression-free survival (Kipps et al., 2015). However, Navitoclax’s clinical application has been limited by dose-dependent thrombocytopenia, primarily due to its inhibition of BCL-XL, which is essential for platelet survival (Khan et al., 2024). To mitigate these adverse effects, researchers have developed NAV-Gal, a prodrug of Navitoclax that is selectively activated by senescence-associated β-galactosidase (SA-β-Gal) within senescent cells, thereby reducing off-target toxicity in non-senescent cells (González-Gualda et al., 2020). Beyond BCL-2 family-targeting senolytics, alternative senolytic strategies have been explored. For example, the combination of dasatinib (a tyrosine kinase inhibitor) and quercetin (a flavonoid antioxidant) has been demonstrated to clear senescent cells effectively, improving cardiovascular function and lifespan in aged mice (Paez-Ribes et al., 2019). This combination therapy has also shown efficacy in patients with idiopathic pulmonary fibrosis (Singh et al., 2025). Additionally, mTOR inhibitors such as AZD8055 and temsirolimus have been investigated as senolytic agents, acting by modulating SASP and enhancing the immune-mediated clearance of senescent cells (Kucheryavenko et al., 2019).

Despite its potential, senolytic therapy faces several challenges. First, the development of novel senolytic agents with improved selectivity and reduced toxicity is crucial. Many current senolytic compounds were initially developed for aging-related diseases, and their efficacy across different cancer subtypes remains variable. Second, the lack of specific senescence biomarkers complicates the identification and monitoring of senescent cells in clinical settings, making it difficult to assess therapeutic responses. Third, tumor heterogeneity poses a significant challenge, as different cancer cell subpopulations may exhibit varying degrees of sensitivity to senolytic therapy. The future development of next-generation senolytic drugs is expected to enhance therapeutic precision by targeting senescence-specific vulnerabilities while minimizing adverse effects. In general, senolytics therapy provides a new perspective for cancer therapy. Although there are still many problems to be solved, its potential cannot be ignored. The application of senolytic therapy in cancer treatment is a complex yet promising field. It requires an in-depth understanding of senescence biology alongside the development of novel senolytic agents and targeted delivery technologies. As research progresses, senolytic therapy is expected to become an integral component of cancer treatment, offering new opportunities to overcome therapy resistance and improve patient outcomes.

#### 4.1.4 Senomorphics: Modulating senescent cell function in cancer therapy

In contrast to senolytics that remove senescent cells, senomorphic strategies aim to modulate the functional phenotype of senescent cells, particularly by suppressing the detrimental components of the SASP, without necessarily inducing cell death. This approach focuses on mitigating the pro-tumorigenic influence of senescent cells on the TME by altering their secretory profile and other functions. Senomorphics primarily target signaling hubs governing SASP amplification, including NF-κB, p38 MAPK, mTOR, and JAK/STAT signaling (Dong Z. et al., 2024), which are central regulators of SASP factor expression. NF-κB signaling serves as the master regulator of SASP-associated inflammation. Metformin inhibits nuclear translocation of NF-κB components and their transactivation at target promoters, thereby suppressing SASP factor expression—mechanistically explaining its anti-aging and anti-tumor effects in diabetic models and patients (Guo et al., 2024). Rapamycin, an mTOR inhibitor, context-dependently induces senescence (Blagosklonny, 2022), diminishes NF-κB activity (Laberge et al., 2015), suppresses pro-inflammatory SASP translationally, and attenuates senescence-mediated tumor promotion (Wang et al., 2017). Hypoxia mimetics (e.g., roxadustat) attenuate mTOR activation to interfere with SASP expression, reducing treatment-induced adverse effects in preclinical models (Schmitt et al., 2022). Similarly, p38 pathway inhibitors suppress SASP, mitigating bone loss and metastasis in therapy-induced senescent breast cancer (Robert et al., 2024). A promising countermeasure involves the use of ginsenoside Rh2, derived from ginseng, which has shown potential in mitigating these effects. Specifically, Rh2, at a concentration of 20 μg/mL, has been shown to reduce the number of senescent breast epithelial cells induced by ROS and the NF-κB/IL-8 pathway. This reduction extends the survival of normal breast epithelial cells and decreases SASP secretion, thereby inhibiting the growth of MCF-7-GFP cells (Hou J. et al., 2020). In prostate cancer, the disruption of sGC (soluble guanylate cyclase) signaling inhibits oxidative stress-induced senescence in response to androgen deprivation (Zhang et al., 2023). Riociguat, an sGC agonist, has been identified as a potential agent for reverse resistance in cancer cells. Oxidative stress-induced aging phenotypes during chemotherapy are typically associated with poor prognosis, chemoresistance, and reduced survival rates in cancer patients. Senescent cancer cells evade the anticancer effects of chemotherapy by re-entering the cell cycle (Lagopati et al., 2021). MHY1485, a mTOR activator (at 10 μM), increases oxidative stress and senescence in radiation-treated cells, promoting increased radiosensitivity compared to radiation therapy alone (Sun L. et al., 2021). Additionally, in the case of 5-fluorouracil, Metformin has been shown to significantly reduce intestinal injury induced by the mTOR/p70S6K pathway, offering therapeutic potential to minimize oxidative stress-induced senescence in normal cells (Xia et al., 2022).

More importantly, senomorphic agents exert profound remodeling effects on the TME by strategically reprogramming the SASP. Through selective suppression of key SASP components like IL-6 and VEGF, senomorphics attenuate immunosuppressive networks—notably by alleviating TGF-β-mediated inhibition of cytotoxic T cells and NK cells—while concurrently reducing pro-angiogenic signaling via VEGF downregulation and impeding metastatic niche formation through diminished MMP activity (Alqahtani et al., 2025). This multipronged modulation resolves chronic inflammation by lowering IL-6/IL-8-driven neutrophil infiltration and macrophage M2 polarization, thereby converting the TME from a tumor-permissive state to one that restricts malignant progression (Zhao et al., 2021).

Senomorphic strategies offer distinct advantages: they may avoid potential complications associated with cell clearance and could provide a more nuanced control over the diverse effects of SASP. By specifically targeting harmful SASP components, they aim to convert the TME from pro-tumorigenic and immunosuppressive towards a less supportive state for cancer growth and progression. However, key challenges remain. Senomorphic effects are typically reversible upon drug withdrawal, necessitating continuous treatment. Achieving complete and selective suppression of detrimental SASP factors while preserving potentially beneficial ones is difficult. Furthermore, senomorphics do not reduce the senescent cell burden, leaving the potential for residual detrimental effects or phenotypic reversion. Developing highly selective senomorphics and understanding the long-term consequences of SASP modulation are critical areas for future research. Ultimately, combining senolytics (to eliminate the source) and senomorphics (to immediately suppress the harmful output) represents a promising synergistic approach to comprehensively tackle the problem of therapy-induced senescence and its detrimental impact on the TME and treatment outcomes (Lelarge et al., 2024).

### 4.2 Redox dyshomeostasis and senescence in emerging therapy

#### 4.2.1 An emerging field of redox biology: Treatment-induced senescence escape

Therapy-induced senescence (TIS) is typically defined as a state of growth arrest following radiotherapy or chemotherapy. However, an emerging concept, aging and escape, has recently gained attention, challenging conventional understanding in this field. An early study demonstrated that clinical concentrations of Adriamycin induced senescence in MCF-7 breast cancer cells (p53 WT, p16 NULL) (Bajtai et al., 2025). A small subpopulation of these cells escaped sustained growth arrest, potentially contributing to chemoresistance in response to senescence-inducing therapies (Gillespie et al., 2025). In 2005, Daniel Wu’s team first reported that a subpopulation of H1299 non-small cell lung cancer cells, lacking TIS p53 and p16, escaped replication arrest and re-entered the cell cycle. These escaped cells exhibited abnormally high levels of cyclin-dependent kinase (Cdc2/Cdk1) (Roberson et al., 2005). Further studies have identified that in colorectal cancer, treatment with Baveromycin A1 (Baf1A), an authenticated inhibitor, restores the proliferation capacity of senescent cells (Ciardiello et al., 2025). These cells exhibited enhanced stem-like properties, including upregulation of NANOG expression, expansion of the CD24^−^ cell population, and increased tumor formation (Alizadeh et al., 2025). Recent research has demonstrated that IFN-γ signaling induced by viral mimicry can promote senescence escape by activating ERK5 (Gureghian et al., 2023). Additionally, B-cell lymphoma samples from Eμ-Myc transgenic mice demonstrated significant activation of stem cell characteristics in both aging and non-aging B-cell populations, which correlated with activation of the WNT signaling pathway. This phenomenon was also observed in cell groups that re-entered the cell cycle after escaping senescence, exhibiting notable stem-like features (Milanovic et al., 2018). These findings suggest that, after senescence-induced growth arrest is reversed, key features such as enhanced stemness remain intact. Notably, senescence evasion cells induced by cancer therapies also retain aging-related chromatin marks, providing strong evidence that senescence-related genetic programming can be inherited, forming senescent scars (Martínez-Zamudio et al., 2023). The oxidative environment in senescence evasion cells differs significantly from that of senescent cells. Specifically, aging cells typically exhibit elevated reactive oxygen species (ROS) and an oxidative environment (Maldonado et al., 2023), whereas our study on breast cancer cells escaping TIS revealed a low ROS environment, resembling that of cancer stem cells (Bajtai et al., 2025). In this low ROS environment, we observed the expression of superoxide dismutase (SOD1/2) and glutathione peroxidase (GPx1/2), as well as activation of the Nrf2 signaling pathway, essential for maintaining the redox balance (Ngo and Duennwald, 2022)). Furthermore, the stability of Nrf2 in TIS escape cells was found to be mediated by p21, a critical factor for tumor stem cell enrichment (Robert et al., 2024).

Studies suggest that polyploid cells may represent a state of senescence evasion, with Rajaraman et al. defining this phenomenon as neurosis—a process by which cells with rejuvenated capabilities are generated through non-synchronized cell division (Dudkowska et al., 2021). In many studies, it has been found that aging multi-time cells generate single-core cells through germination or a non-synchronized division mechanism (He X. et al., 2020; Park et al., 2024). Such cells often display increased aggressiveness and, in some cases, enhanced resistance to genotoxic therapies (Shokrzadeh et al., 2021; Feng et al., 2022). Moreover, aging polyploid cells can promote the survival of adjacent tumor cells by releasing various factors, including VEGF and MIF, which are induced by high ROS levels (Duan et al., 2024). Currently, the mechanisms underlying oxidative stress and changes in redox homeostasis during aging in cells remain incompletely understood. It is hypothesized that the formation of a low ROS environment in senescence evasion cells may be closely related to cell cycle regulation mechanisms, which differ from those in senescent cells (Evangelou et al., 2023). This redox reprogramming could play a critical role in triggering senescence escape and early cell cycle re-entry (Liu L. et al., 2025). Therefore, dynamic analysis of redox changes during senescence evasion transitions is crucial to elucidate this phenomenon. Furthermore, integrating multidisciplinary approaches to investigate the genetic marks and metabolic pathways involved in redox modulation will be pivotal in advancing our understanding of senescence evasion mechanisms. These discoveries are of significant importance for addressing aging-related resistance in cancer therapy and offer valuable insights into the plasticity of cancer cells.

#### 4.2.2 Nanotechnology-mediated redox dyshomeostasis: a pivotal approach for advancing high-efficiency oncological therapies

Compared to traditional chemotherapy agents, nanomaterials exhibit unique physicochemical properties, including passive targeting capabilities, drug delivery functions, and a propensity to engage in electron transfer processes. These unique properties enable nanomaterials to be precisely engineered to respond to endogenous conditions, such as low pH and hypoxia, or to external stimuli, including light irradiation and ultrasound. This capability enables the modulation of tumor cell redox balance, thereby enhancing therapeutic efficacy (Guo et al., 2025).

##### 4.2.2.1 Regulation of oxidizing species to realize RDH

Oxidizing species primarily include reactive oxygen species (ROS) and reactive nitrogen species (RNS). ROS are highly reactive oxygen-containing molecules, including superoxide anions (O2•−), hydrogen peroxide (H2O2), and hydroxyl radicals (•OH). RNS, which mainly consists of nitric oxide (NO) and peroxynitrite (ONOO•−), also plays a significant role in oxidative stress responses (Sies et al., 2022). Among various oxidizing species, H_2_O_2_ is particularly notable due to its stability and ability to diffuse within cells, making it a key ROS mediator in redox regulation. As a critical intermediate in mammalian oxygen metabolism, H_2_O_2_ can rapidly convert into multiple ROS, thereby modulating intracellular oxidative stress (Jose et al., 2024). Given its central role, H_2_O_2_ has emerged as a primary target in ROS-mediated therapeutic strategies. Precise H_2_O_2_ level modulation is crucial for disrupting cellular redox homeostasis and inducing oxidative stress-mediated cancer cell death (Zaher et al., 2024). In recent years, researchers have developed nanomaterials capable of implementing redox dyshomeostasis (RDH) strategies by enhancing H_2_O_2_ production, thereby improving cancer treatment efficacy. H_2_O_2_ level augmentation can be achieved through two primary mechanisms: biological synthesis and chemical pathways (Glorieux et al., 2024). In the biological synthesis approach, nanomaterials enhance enzyme-mediated H_2_O_2_ generation, thereby disrupting the redox balance of tumor cells. For instance, FePt-NP2 nanoparticles, activated by cisplatin, stimulate NADPH oxidase (NOX) to convert O_2_ into superoxide anions, which subsequently generate H_2_O_2_ and intensify tumor oxidative stress, ultimately promoting cancer cell apoptosis (Yu et al., 2021). Similarly, GOx-Fe3O4@DMSNs nano-catalysts utilize glucose oxidase (GOX) to convert glucose into H_2_O_2_, thereby amplifying intracellular oxidative stress, enhancing the Fenton reaction, and improving cancer therapy outcomes (Wang et al., 2025). Regulating lactic acid and energy metabolism is another mechanism that causes H_2_O_2_ (Rabinowitz and Enerbäck, 2020; Sun M. et al., 2025). At the same time, hydrogen peroxide levels can be increased by sending hydrogen peroxide directly to the tumor cells, but this method is still immature, and the hydrogen peroxide delivered can easily be broken down by intracellular hydrogen peroxide. Therefore, metal peroxide can specifically respond to the microenvironment of acidic tumors, producing a large amount of H_2_O_2_ in the tumor site, which may destroy the oxidation and restoration of the tumor and eventually enhance the sensitivity of tumor cells to other treatments. Chen et al. reported a method for making copper-oxygen nano (CP) (Tang et al., 2020). Copper-oxygen nano (CP) is decomposed in an acidic environment of the inner cut/lymphatic body, which allows the release of Fenton-catalyzed Cu^2+^ and H_2_O_2_(Xiao et al., 2025a). At the same time, Sumid Cu^2+^ induced CDT, changing the balance of metal ion content can induce an intracellular response and even cell death. This method has been used to treat tumors and is defined as ion interference therapy (IIT) (Tang et al., 2020). The combination of CDT and IIT into further material design and corresponding tumor therapy opened a new door.

##### 4.2.2.2 Regulation of reducing species to realize RDH

To counteract oxidative stress, cancer cells rely on a robust antioxidant defense system that maintains intracellular redox equilibrium through the production of reducing agents (Chen et al., 2025). Reducing agents, such as glutathione (GSH), serve as key antioxidants by neutralizing ROS, thereby attenuating the cytotoxic effects of ROS-based cancer therapies. Thus, targeting GSH depletion has become a prominent strategy in RDH-based therapies (Wu S. et al., 2022; Wu Y. et al., 2022; Roy and Paira, 2024). GSH plays a pivotal role in neutralizing ROS and protecting cancer cells from oxidative damage. As a result, GSH depletion strategies have been widely explored to enhance tumor sensitivity to oxidative stress-mediated treatments. Various nanomaterials have been designed to suppress GSH biosynthesis or deplete intracellular GSH levels, thereby disrupting redox homeostasis and enhancing therapeutic efficacy (Niu et al., 2021; Xiong et al., 2021). These approaches can be classified into two primary categories: 1. Inhibiting GSH biosynthesis to reduce intracellular GSH levels, thereby promoting oxidative stress. For instance, Wang et al. developed a near-infrared (NIR) thermo-responsive liposomal nano-antagonist (PLAN), incorporating indocyanine green (ICG) and buthionine sulfoximine (BSO). BSO inhibits GSH biosynthesis, thereby disrupting redox homeostasis and enhancing ICG-mediated photodynamic therapy (PDT) efficacy (Sun et al., 2020). 2. Directly depleting intracellular GSH levels through catalytic consumption. This includes oxidizing agents and electrophiles that react with GSH, converting it into inactive byproducts and promoting oxidative stress-induced tumor cell apoptosis (Hu et al., 2019; Fu et al., 2021). Chen et al. constructed MnO_2_-based mesoporous silica nanoparticles (MS@MnO_2_ NP). Once taken up by cancer cells, the MnO_2_ was used to oxidize GSH to accumulate H_2_O_2_, which led to RDH, ultimately enhancing Mn^2+^-mediated CDT (Lin et al., 2018). Furthermore, the CoFe2O4 nanoplatform (CFAP), functionalized with polyethylene glycol (PEG)-coated gold nanoparticles, effectively depletes GSH, disrupts intracellular redox balance, and induces immunogenic cell death, exhibiting promising clinical potential for solid tumor treatment (Zeng et al., 2024). Importantly, besides GSH, the thioredoxin/thioredoxin reductase (Trx/TrxR) system in cancer is another important antioxidant defense system (Seitz et al., 2024). It has been reported that Au nanoclusters could effectively inhibit TrxR in the tumor cell cytoplasm (Xiao et al., 2025b). Gao et al. constructed spatiotemporal controllable liposomal nanocomposites co-loaded with Au nanoclusters and photosensitizer Chlorine 6 (Ce6). Au nanoclusters effectively inhibited TrxR to induce RDH, which enhanced the Ce6-mediated PDT efficacy (Gao et al., 2017).

##### 4.2.2.3 Innovative nanomaterial-based therapeutic strategies

The integration of nanotechnology into oncology has revolutionized therapeutic design, particularly in overcoming the challenges of tumor drug resistance driven by redox dyshomeostasis and therapy-induced senescence. Recent advances in nanomaterials offer unprecedented opportunities to manipulate intracellular redox environments, eliminate senescent tumor cells, and re-sensitize drug-resistant populations. By engineering nanoplatforms that can dual-target redox and senescence pathways, researchers are developing highly specific, responsive, and multifunctional systems to reshape the tumor microenvironment and enhance therapeutic outcomes. Recent advances in nanotechnology-based tumor therapies have introduced multifunctional enzymes, such as IB@Fe-ZIF8@PDFA, which integrate peroxidase (POD), GSH oxidase (GSH-OXD), and NADH oxidase (NADH-OXD) to potentiate ferroptosis under laser irradiation, demonstrating enhanced anticancer activity and reduced metastasis (Qin et al., 2024). Similarly, a biodegradable tumor-targeting nanocarrier designed to release Fenton-like Cu2+/Cu enhances GSH depletion, inhibits catalase (CAT) activity, and promotes the generation of hydroxyl radicals (•OH), ultimately leading to tumor apoptosis (Lu et al., 2022). Additionally, dual-metal PtPd atomic clusters (BAC) have been shown to induce apoptosis, alleviate local hypoxia, and promote ROS-driven lipid peroxidation, thereby enhancing ferroptosis and suppressing tumor metastasis (Wang X. et al., 2024). Jun Lin and others made a multifunctional nano-vaccine based on a black melonhole titanium dioxide (BMT) based on L-arginine (LA). By using ultrasound, BMT and LA generate NO, which not only induces RDH but also induces apoptosis through the DNA double-strand break (Wang M. et al., 2021). All of this ultimately enhances the immunotherapy of the PD-L1 antibody, further inhibiting metastatic tumor growth and achieving a more effective anti-tumor effect, as well as inhibiting tumor metastasis (Wang M. et al., 2021). These innovative nanomaterial-based approaches highlight the potential of precisely regulating oxidative and reductive species to induce redox dyshomeostasis in cancer cells. By leveraging these strategies, researchers can develop next-generation therapeutics that significantly improve treatment efficacy while minimizing off-target effects, thereby paving the way for highly efficient oncological therapies.

Beyond traditional polymers and metal-based systems, emerging nanomaterials such as metal-organic frameworks (MOFs) and DNA origami nanostructures have shown promise for precisely manipulating redox and senescence-related signals. MOFs, with their customizable porosity and catalytic capabilities, have been utilized to deliver both ROS-generating and ROS-scavenging agents in a tumor-specific manner (He H. et al., 2020; Zhao et al., 2022). Meanwhile, DNA nanostructures are being investigated for their programmability in assembling stimuli-responsive senolytic payloads and siRNAs targeting redox enzymes (Luo et al., 2022). In summary, nanotechnology offers a transformative platform for targeting redox dyshomeostasis and senescence in drug-resistant tumors strategically. By developing responsive, multifunctional nanomaterials that orchestrate redox modulation, senescence induction, and immune activation, the field moves closer to achieving personalized, durable cancer therapy.

## 5 Conclusion and perspective

Tumor drug resistance remains a formidable challenge in oncology, driven in part by the complex, context-dependent interplay between redox dyshomeostasis and cellular senescence. This review synthesizes evidence showing that oxidative stress-induced senescence initially serves as a tumor-suppressive barrier during early carcinogenesis but paradoxically evolves into a key mediator of resistance via the persistent SASP. The SASP fosters a pro-tumorigenic microenvironment that enables immune evasion, angiogenesis, and treatment failure. Consequently, therapeutic strategies targeting this axis—specifically senolytics to eliminate senescent cells and SASP inhibitors to neutralize their detrimental secretions—offer substantial promise for overcoming resistance. However, translating these strategies to the clinic faces substantial challenges that require critical evaluation. Senolytics face critical limitations, including insufficient specificity for heterogeneous senescent cell populations, which causes off-target cytotoxicity against vital non-senescent cells (e.g., stem/progenitor cells) and potential disruption of beneficial senescence functions in tissue repair; dose-limiting toxicities (e.g., thrombocytopenia); and unforeseen immune complications. The removal of immunomodulatory senescent cells by senolytics may inadvertently dampen anti-tumor immune responses. Similarly, SASP inhibition is complicated by the functional heterogeneity and temporal dynamics of the secretome. Broad suppression risks blocking beneficial immune-stimulating factors alongside pro-tumorigenic cytokines (e.g., IL-6, TGF-β), creating an immune modulation dilemma (Tahmasebi et al., 2023), whereas achieving tumor-specific delivery to avoid systemic immunosuppression remains a significant pharmacological challenge. Looking forward, overcoming these limitations and harnessing the therapeutic potential of the redox-senescence axis requires focused efforts on several key fronts: First, deciphering the precise molecular and contextual determinants (tumor type/stage, microenvironment) that govern whether this axis suppresses or promotes cancer is essential. Second, developing strategies for spatiotemporally precise intervention is critical to selectively eliminate or modulate detrimental senescence while preserving its beneficial functions. Third, identifying and validating robust multimodal biomarkers (e.g., combinations of p16^INK4a^, Lamin B1, SASP factors, and redox signatures such as mitochondrial ROS) for patient stratification and response monitoring is crucial for personalized therapy (Lv et al., 2024; Bajtai et al., 2025). Fourth, advanced nanotechnology platforms provide compelling solutions, enabling ROS-responsive drug release, targeted delivery of senolytics/SASP modulators to specific cell populations or organelles, and controlled induction of RDH to enhance therapeutic efficacy while minimizing off-target effects, as demonstrated in preclinical models (Chauhan et al., 2024; Hu et al., 2024). Finally, understanding emerging phenomena such as treatment-induced senescence escape is vital for preventing therapeutic failure.

In conclusion, although targeting the redox-senescence interplay offers a multifaceted strategy to combat drug resistance, its successful clinical translation hinges on acknowledging and rigorously addressing the inherent biological complexities and translational barriers, especially those associated with senolytics and SASP modulation. Future progress requires integrating fundamental mechanistic insights with innovative engineering approaches such as nanomaterials and gene editing, and biomarker-driven clinical trials to translate this promising paradigm into safe, effective, and personalized oncological therapies.
